# Influence of Vascular Variant of the Posterior Cerebral Artery (PCA) on Cerebral Blood Flow, Vascular Response to CO_2_ and Static Functional Connectivity

**DOI:** 10.1371/journal.pone.0161121

**Published:** 2016-08-17

**Authors:** Kirsten Emmert, Daniela Zöller, Maria Giulia Preti, Dimitri Van De Ville, Panteleimon Giannakopoulos, Sven Haller

**Affiliations:** 1 Faculty of Medicine, University of Geneva, Geneva, Switzerland; 2 Medical Image Processing Laboratory, Institute of Bioengineering, Ecole Polytechnique Fédérale de Lausanne (EPFL), Geneva, Switzerland; 3 Office Médico-Pédagogique, Department of Psychiatry, University of Geneva, Geneva, Switzerland; 4 Department of Psychiatry, University of Geneva, Geneva, Switzerland; 5 Affidea Centre de Diagnostic Radiologique de Carouge CDRC, Carouge, Switzerland; 6 Department of Surgical Sciences, Radiology, Uppsala University, Uppsala, Sweden; Ghent University, BELGIUM

## Abstract

**Introduction:**

The fetal origin of the posterior cerebral artery (fPCA) is a frequent vascular variant in 11–29% of the population. For the fPCA, blood flow in the PCA originates from the anterior instead of the posterior circulation. We tested whether this blood supply variant impacts the cerebral blood flow assessed by arterial spin labeling (ASL), cerebrovascular reserve as well as resting-state static functional connectivity (sFC) in the sense of a systematic confound.

**Methods:**

The study included 385 healthy, elderly subjects (mean age: 74.18 years [range: 68.9–90.4]; 243 female). Participants were classified into normal vascular supply (n = 296, *76*.*88%*), right fetal origin (n = 23, *5*.*97%*), left fetal origin (n = 16, *4*.*16%*), bilateral fetal origin (n = 4, *1*.*04%*), and intermediate (n = 46, *11*.*95%*, *excluded from further analysis*) groups. ASL-derived relative cerebral blood flow (relCBF) maps and cerebrovascular reserve (CVR) maps derived from a CO_2_ challenge with blocks of 7% CO_2_ were compared. Additionally, sFC between 90 regions of interest (ROIs) was compared between the groups.

**Results:**

CVR was significantly reduced in subjects with ipsilateral fPCA, most prominently in the temporal lobe. ASL yielded a non-significant trend towards reduced relCBF in bilateral posterior watershed areas. In contrast, conventional atlas-based sFC did not differ between groups.

**Conclusions:**

In conclusion, fPCA presence may bias the assessment of cerebrovascular reserve by reducing the response to CO_2_. In contrast, its effect on ASL-assessed baseline perfusion was marginal. Moreover, fPCA presence did not systematically impact resting-state sFC. Taken together, this data implies that perfusion variables should take into account the vascularization patterns.

## Introduction

In most healthy humans the basilar artery feeds the posterior cerebral artery (PCA) via the P1 segment. However, in about 11–29% of all healthy humans the PCA is not supplied by the P1 segment but the posterior communicating artery (PcomA) connecting the middle cerebral artery (MCA) and the PCA [1]. There are two main consequences of this norm-variant referred to as the fetal PCA (fPCA). First, the leptomeningeal vessels cannot be developed between the anterior and posterior circulation [1]. Second, a larger brain area is supplied by the internal carotid artery that feeds the anterior cerebral artery (ACA), MCA and, in this variant, the PCA via the PcomA. The fPCA was chosen as a target for this study as it is the most common vascular variant of the circle of Willis. At the same time, it is a potentially relevant vascular variant because in the presence of this variant, the PCA territory is supplied via the anterior instead of the posterior circulation. It has been shown that unilateral fPCA results in a left-right asymmetry of perfusion images when assessing macrovascular transit effects [2].

Our objective was to assess the three basic parameters that might be confounded by this vascular norm-variant of the PCA: basal cerebral perfusion, vascular reserve, and resting-state functional connectivity.

The cerebral blood flow (CBF) was measured by arterial spin labeling (ASL). ASL uses arterial blood water as an intrinsic tracer to non-invasively measure tissue perfusion. When the MCA and PCA are both fed from the carotid artery, one might expect changes in cerebral perfusion in either of these two vascular territories and especially the watershed zone between the MCA and PCA.

The cerebrovascular reactivity (CVR) describes the response of the cerebral vasculature to vasodilatory stimuli. CVR can easily be assessed with functional Magnetic Resonance Imaging (fMRI) by inhalation of CO_2_ enriched air during the acquisition, causing a global vasodilatation in the brain measurable by the blood oxygenation level dependent (BOLD) fMRI contrast in comparison to breathing of normal air. The CVR can vary in amplitude and slope of the response [3]. In case of the fPCA, the slope of the onset might be affected, due to different flow velocities. A reduced CVR often occurs in internal carotid artery stenosis or occlusion [4]. Interestingly, after carotid revascularization the CVR recovers and an initial high CVR reduction seems to increase the risk of new peri-interventional infarcts [5]. In addition, the CVR is temporarily reduced in an area when it is recruited for an ongoing task [6].

Finally, fMRI does not directly assess neuronal activation, but the vascular response due to the neurovascular coupling [7]. Consequently, the presence of the fetal origin of the PCA might affect the cerebral auto regulation and result in alterations of the fMRI response in the sense of a systematic confound. In addition, the PCA variant might impact the static functional connectivity (sFC) during resting state fMRI between MCA and PCA and especially the connectivity between the MCA-PCA watershed zone and the MCA or PCA. The sFC measure is based on the correlation of BOLD time courses between these two areas with high sFC values reflecting a large amount of information flow between them. Due to the fact that there is a ceiling of the BOLD response [6], local changes of the vascular supply may impact resting-state fMRI fluctuations in areas closer to the saturation level than under resting conditions. It is thus possible that the presence of this frequent vascular variant impacts functional connectivity estimated based on fMRI.

In our study, we show that variation of the PCA influences vascular reserve while basal cerebral perfusion is only marginally (non-significantly) influenced and resting-state functional connectivity does not seem to be affected significantly.

## Material and Methods

### Participants

385 elderly, healthy participants (mean age: 74.18 ± 4.10, 243 female) were recruited at two medical centers in Switzerland (Geneva and Lausanne). They gave written informed consent to participate in this study that was approved by the Commission cantonale d'éthique de la recherche Genève (cantonal ethics committee Geneva) and has been performed in accordance with the ethical standards prescribed in the 1964 Declaration of Helsinki and its later amendments. Here, we look at an interesting vascular variant and its effects on different MRI measures. Participants were classified into seven different groups depending on their PCA variant: standard variant (fed from the basilar artery, n = 296, *76*.*88%*), fetal origin on the right (n = 23, *5*.*97%*), fetal origin on the left (n = 16, *4*.*16%*), bilateral fetal origin (n = 4, *1*.*04%*), and one group each for right (n = 25, *6*.*49%*), left (n = 15, *3*.*90%*) or bilateral (n = 6, *1*.*56%*) variants that were intermediate (with comparable diameters of the basilar artery and PComA connection, excluded from further analyses, for details see Table 1). The vascular system was analyzed by KE (3 years of experience in MR imaging) and SH in consensus (14 years of experience in neuroradiology). The PCA was considered of fetal origin when the P1 segment was clearly inferior in diameter when compared to PCA. The PCA was considered normal, when the P1 segment was clearly dominant over the PComA. Cases with PComA and P1 segments of similar diameters were classified as intermediate and excluded from further analyses as they cannot be categorized clearly into fPCA or normal PCA. This analysis was performed based on additionally acquired susceptibility weighted imaging (SWI) and T2 images.

**Table 1:** Demographics of participants included in our study (age: mean +/- standard deviation).

Variable	Standard PCA	Right variant	Left variant	Bilateral variant	Right intermed.	Left intermed.	Bilateral intermed.
n	296	23	16	4	25	15	6
Age	74.05+/- 4.21	74.95+/- 3.65	74.44+/- 3.71	71.5+/- 1.29	74.88+/- 3.46	74.53+/- 3.81	74.67+/- 5.16
Sex (% female)	63.51	63.64	62.50	50.00	68.00	60.00	50.00

For analysis of unilateral changes due to fPCA, left variants were mirrored and bilateral variants were excluded.

### MRI Data Acquisition

ASL, anatomical and fMRI data was acquired from all participants. Images were acquired on a 3T MRI Scanner (Siemens Tim Trio, Erlangen, Germany) situated in Geneva using a 32-channel head-coil. A high-resolution T1-weighted anatomical scan (magnetization prepared rapid gradient echo (MPRAGE), 256x256 matrix, 176 slices 1mm isotropic, TR = 2300ms, TE = 2.27ms,) was acquired as well as at least one pulsed ASL sequence. For all subjects PICORE (proximal inversion with a control for off-resonance effects, 64x64 matrix, 20 slices 3.44x3.44x6mm voxel, TR = 5000 ms, TE = 21 ms, 71 images) data were acquired.

Functional data acquisition used an echo-planar imaging sequence (EPI, TR = 3000 ms, TE = 30 ms, 45 slices, slice thickness 3 mm without gap, 180 volumes).

SWI images (TR = 28 ms, TE = 20 ms, 128 slices, slice thickness 1 mm, flip angle: 15°) and T2-weighted images (TR = 4000 ms, TE = 105 ms, 30 slices, slice thickness 4 mm with 0.8 mm gap, flip angle: 150°) were used for PCA classification.

### CO_2_ BOLD Paradigm

The functional sequence was used to acquire BOLD data of a nine minute CO_2_ challenge. Synthetic air was mixed with 7% CO_2_ and administered via a nasal cannula alternating with normal air. The paradigm consisted of one minute normal air initially, followed by two minutes of 7% CO_2_ air, two minutes of normal air, two minutes inhaling 7% CO_2_ air again and finally two minutes normal air (1min OFF-2min ON-2min OFF- 2min ON- 2min OFF). This type of CO2 administration has already been successfully used in other studies [3, 5].

### Analysis of MRI Data

#### CO_2_ BOLD response

The BOLD response to the CO_2_ paradigm was assessed with a general linear model analysis using FSL (FSL 5.0.6, FMRIB, Oxford, UK) [8]. The first level analysis used a regressor modeling the ON-OFF block design of the 7% CO_2_ air supply convolved with a filter (as described in [3]). This regressor exhibits exponential behavior, similar to convolving the CO_2_ block design with a gamma function [9]. In the second level analysis, groups were compared using a fixed effects analysis. Regions that resulted as significantly different (standard FSL cluster thresholding, Z = 2.3, p<0.05) between the control and fPCA group were subsequently used for a confirmatory analysis to compare z-scores between the groups.

In order to assess differences in the CO_2_ BOLD response that do not follow the traditional response function, we looked at the time courses of the CO_2_ BOLD response without applying a general linear model to check for potential differences in the shape of the CO_2_ response. Based on visual inspection of the watershed areas a spherical region of interest centered in the watershed area of the MCA and PCA (MNI coordinates +/-59–46–14) was selected and the average time course extracted. Average time courses of the MCA-PCA watershed area (WSA), as well as of the whole brain were normalized (z-scored) and compared between groups to assess possible differences in kinetics of the CO_2_ response. In addition, we designed a regressor that models the on- and offset to assess the CO2 response dynamics. This regressor was previously used in a publication to account for differences in CO2 dynamics between different groups [10] and we therefore used it to compare the on- and offset dynamics between the control and fPCA group.

#### ASL

Analysis of the ASL data was conducted with FSL 5.0.7 (FMRIB Analysis Group, University of Oxford, UK). We assessed baseline cerebral blood flow for subjects without any specific task. Relative cerebral blood flow images were obtained from the MR scanner (in-build processing). Preprocessing of the cerebral blood flow images included normalization and smoothing using a 4-mm Gaussian kernel. FSL randomize was used to compare between PCA variants and non-variants.

#### BOLD sFC

SFC was calculated using the CO_2_ challenge data set after regression of the CO_2_ response. Custom-build MATLAB scripts (MATLAB 2014b, MathWorks, Natick, USA), as well as SPM 8 (UCL, London, UK) including the DPARSF [11] and IBASPM toolboxes [12] were used for analyses. To evaluate sFC, a previously published pipeline [13, 14] was adopted. Preprocessing in SPM 8 included realignment, smoothing using an 8mm Gaussian kernel and co-registration to the high resolution T1 anatomical image.

Then, the segmentation of the T1 image using SPM8’s “New Segment” algorithm resulted in tissue maps for white matter, gray matter and cerebrospinal fluid, used later in the pipeline. The time courses of all fMRI voxels were detrended and nuisance variables were regressed out using the DPARSF toolbox. These included linear and quadratic detrending, six head motion regressors (translation and rotation directions), average cerebrospinal fluid and white matter signal computed from template ROIs registered to the subjects’ functional space and masked with the individual maps obtained from T1 segmentation, as well as a CO_2_ challenge regressor. The CO_2_ regressor [3] was included to eliminate the vasodilatory effect of the CO_2_ from the sFC estimation. The gray matter map was used to mask individual anatomical volumes, subsequently parcellated into 90 cortical and subcortical regions (AAL atlas, [15]) or 12 regions according to vascular territories (custom-made atlas based on known vascular territories and watershed areas WSA as depicted in Radiopaedia.org, courtesy of Dr Frank Gaillard and the IMAIOS online atlas https://www.imaios.com/en/e-Anatomy/Head-and-Neck/Brain-MRI-3D) with the help of a modified version of the IBASPM toolbox. The high resolution parcellations were then resliced to the subject’s functional space and preprocessed BOLD time courses were spatially averaged within the 90/12 atlas regions, yielding one time course per region. In order to limit the analysis to the resting-state frequency range, the averaged time courses were high-pass filtered to remove frequencies below 0.01 Hz in order to reduce artifacts such as scanner drift.

Computation of pairwise Pearson correlations between all ROIs in the atlas results in a 90 × 90 (or 12 x 12) correlation matrix for each subject. For each connection, differences between groups were calculated using a one-way ANOVA.

## Results

### CO_2_ BOLD Response

**Fig 1:**
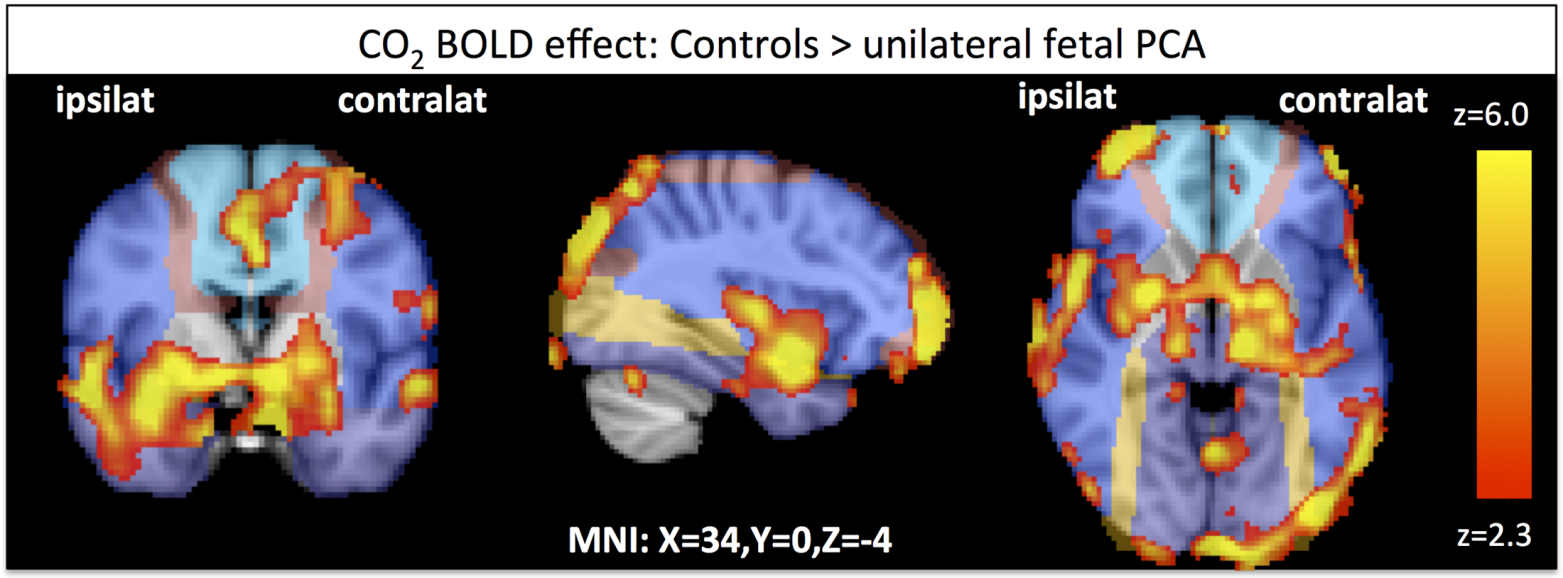
Differences in the CO_2_ response between Controls and unilateral fetal PCA (p<0.05, fixed effects analysis). ACA, MCA and PCA are depicted in shades of blue while watershed areas are colored in yellow-red.

When looking at the fixed effects analysis of the CO_2_ BOLD response of unilateral fPCA compared to controls we observed a significantly decreased BOLD response to CO_2_ predominantly in the ipsilateral temporal part, but also at the basal ganglia, cingulate, frontal and the parieto-occipital regions (Fig 1, S1 and S2 Files).

The mean z-value for the regions shown in the figure is 2.60 (+/- 1.59) for the control group and 2.06 (+/- 1.09) for the fPCA group.

**Fig 2:**
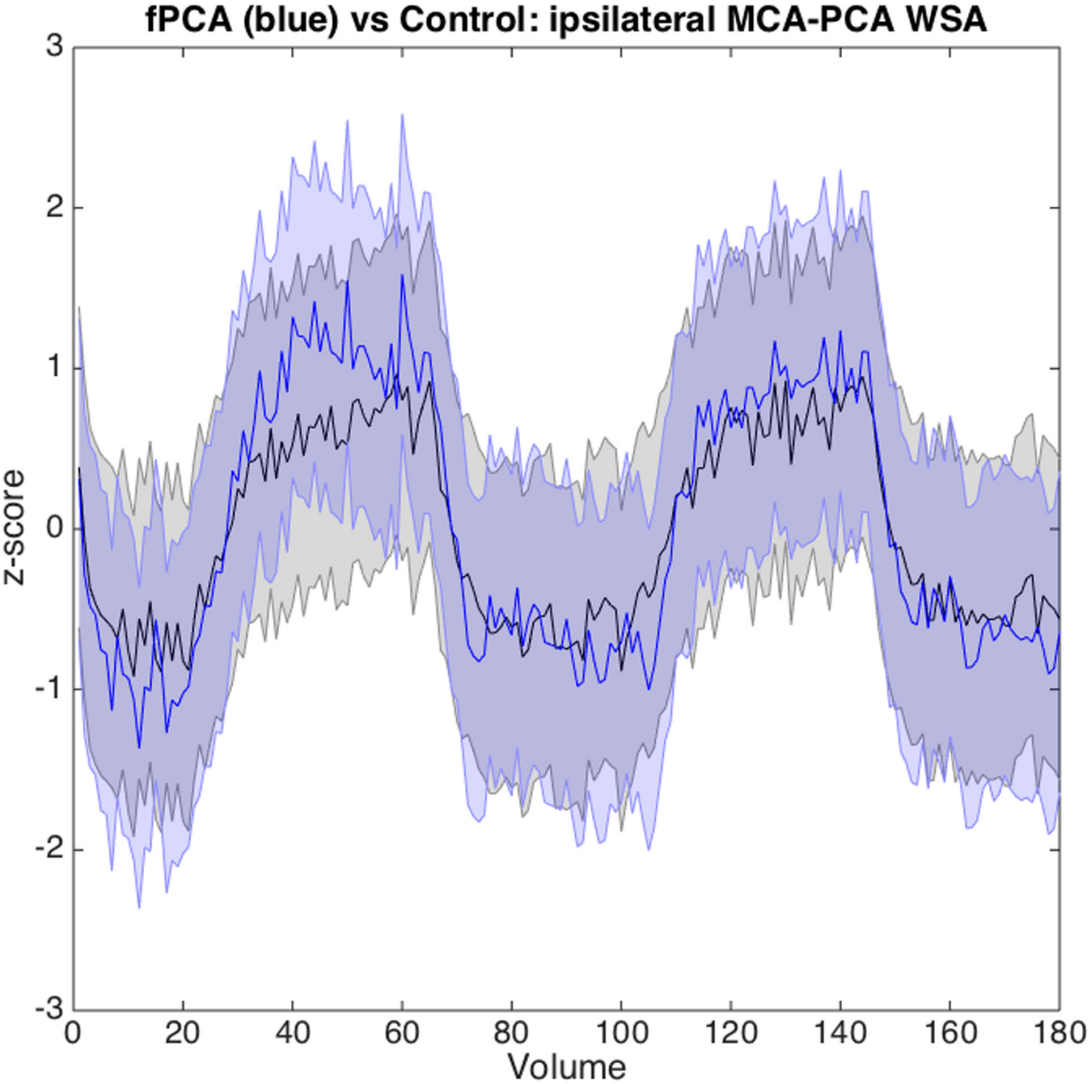
Comparison of the shape of the CO_2_ response of the unilateral fPCA group (blue) and the control group (black) using the ipsilateral MCA-PCA watershed ROI. Time courses are normalized (z-scored) and the shaded error shows one standard deviation.

Subsequently, we analyzed the shape of the average time course of the CO_2_ response using either the whole brain or a region in the MCA-PCA WSA. The fMRI data using the C0_2_ paradigm showed no significant differences in shape of the CO_2_ response, neither for the ROI (Fig 2) nor for the whole brain. Similarly, the comparison of the spatial maps of the regressor modeling the on- and offset between our groups revealed no significant differences.

### ASL Data

**Fig 3:**
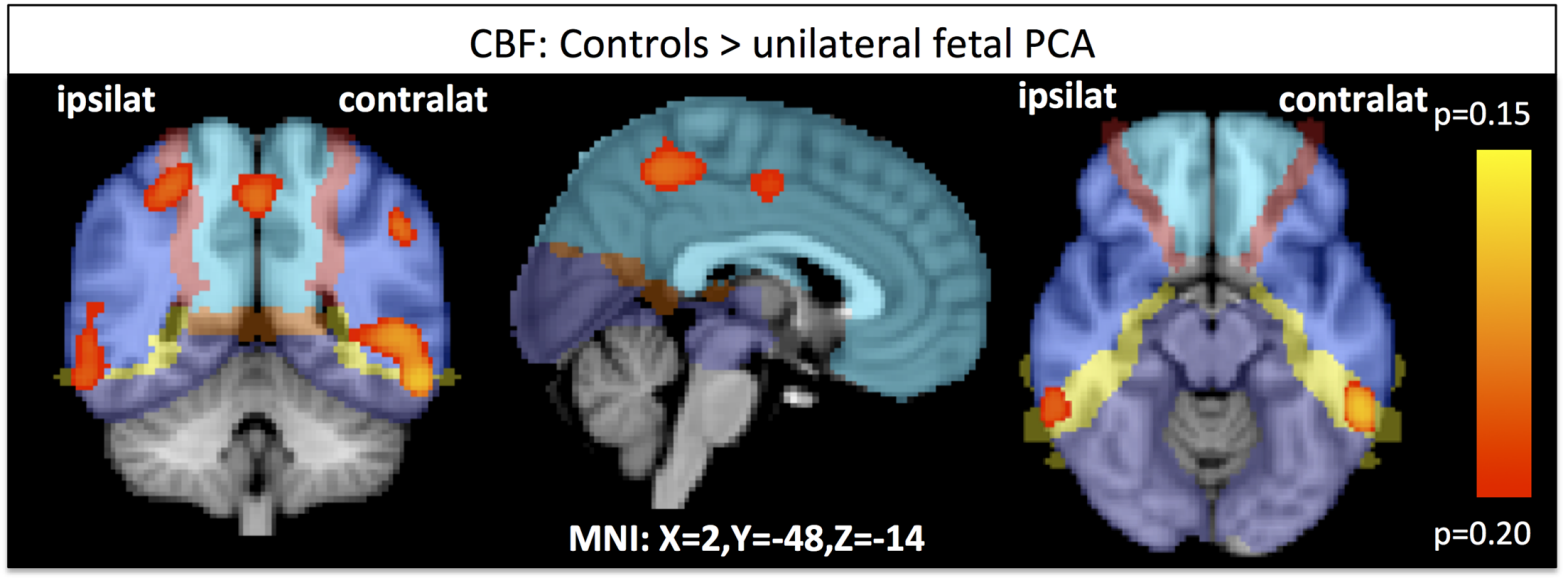
Differences in baseline cerebral blood flow between Controls and unilateral fetal PCA (p<0.2, TFCE-corrected). ACA, MCA and PCA are depicted in shades of blue while watershed areas are colored in yellow-red.

Analysis of the ASL data revealed no significant differences in cerebral blood flow between control subjects and subjects with unilateral fPCA when applying TFCE-correction for multiple comparisons. On a descriptive level (p<0.2 tfce-corrected, p<0.02 uncorrected), we see differences located in or close to the watershed area between MCA and PCA (yellow in Fig 3, S3 File).

The mean CBF differed between the two groups within the activation clusters plotted in Fig 2 (control group: 304.05 +/- 257.21 and the fPCA group: 155.66 +/- 167.30, two-tailed t-test p<0.01). The groups were also different when looking only at the PCC (ROI from activation map shown in Fig 2), an important node in the ageing brain (Control group: 404.97 +/- 298.25; fPCA group: 264.62 +/- 222.16).

### Static Functional Connectivity of BOLD Data

**Fig 4:**
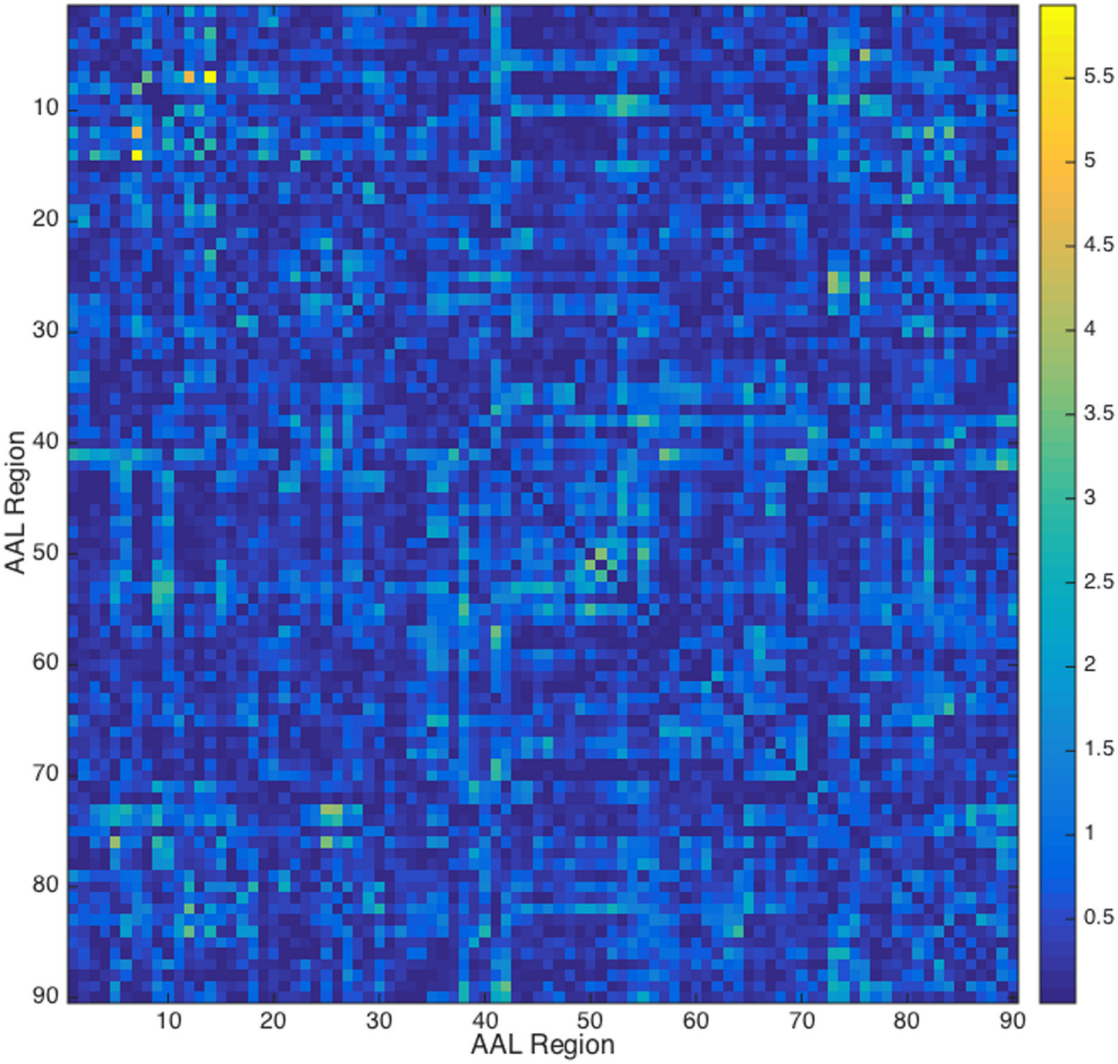
F statistic value of the difference between vascular variants and controls (ANOVA test) for each connection (AAL parcellation). The left middle frontal gyrus (region 7) showed the most significant connectivity differences between groups with the right inferior frontal gyrus, pars opercularis (p(uncor) = 0.009) and the right inferior frontal gyrus, pars triangularis (p(uncor) = 0.003). No connections survived multiple comparison correction.

**Fig 5:**
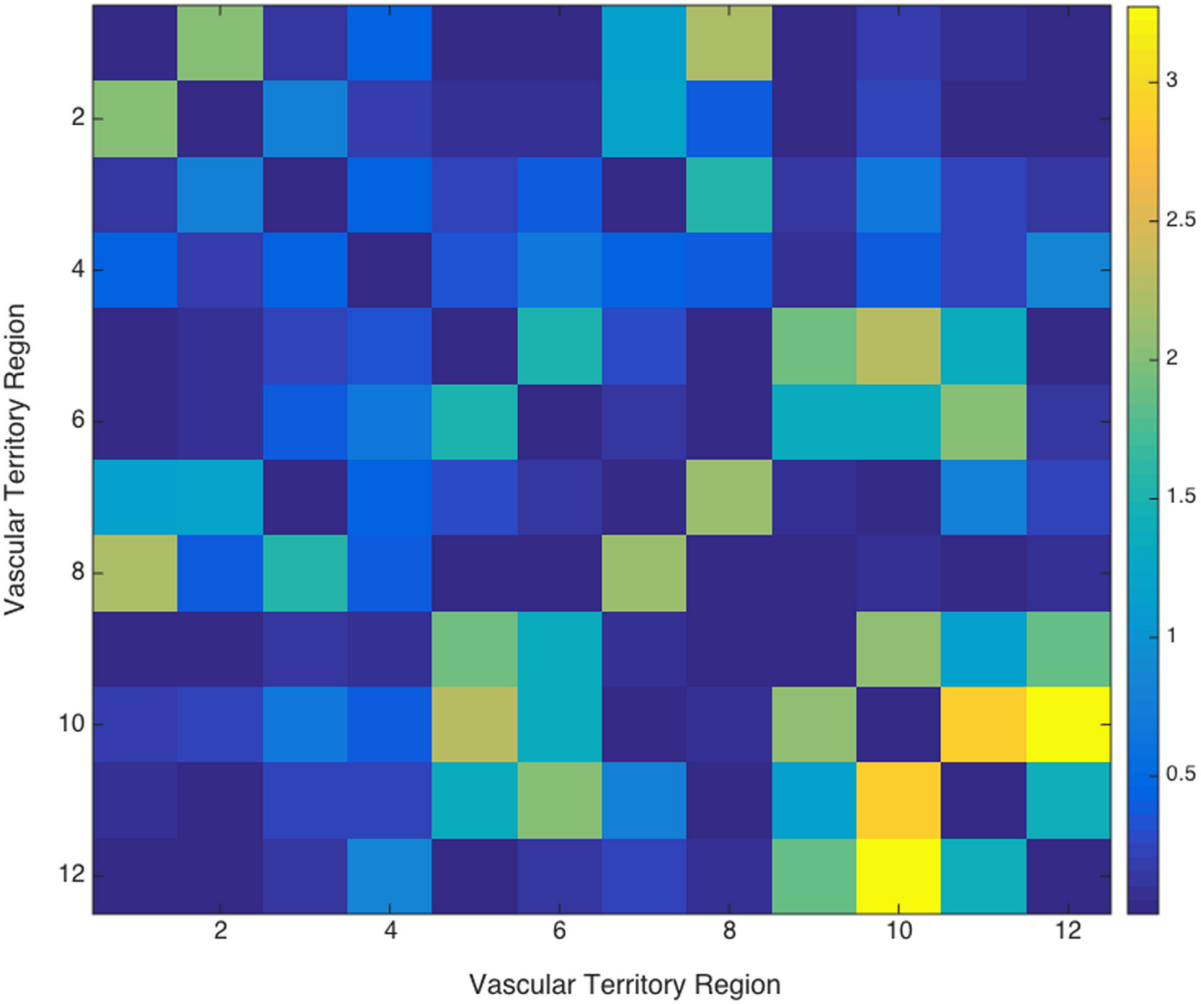
F statistic values of the difference between vascular variants and controls (ANOVA test) for each connection (vascular territory parcellation). The left ACA-PCA watershed area (region 10) showed the most significant connectivity differences between groups with the right (p(uncor) = 0.06) and left ACA-PCA watershed areas(p(uncor) = 0.04). No connections survived multiple comparison correction.

The sFC analysis of the BOLD data revealed no significant differences between vascular variants and controls (maximum F-statistic value: 5.93 (AAL Atlas), p(uncorrected) = 0.003) for both parcellations (AAL atlas, Fig 4 and S4 File, or vascular territory atlas, Fig 5 and S5 File) after multiple comparison correction.

## Discussion

The presence of fPCA significantly reduces the vascular reserve most pronounced in the ipsilateral temporal lobe. The effect on baseline perfusion assessed using ASL is not significant. On a descriptive level we see a non-significant decrease of the CBF most pronounced in the posterior watershed areas. Moreover, the fPCA does not systematically impact resting-state functional connectivity.

### CO_2_ BOLD Response (Vascular Reserve)

We showed that the CO_2_ BOLD response is reduced in the fPCA group in a number of regions including the ipsilateral temporal lobe, the cingulate and the basal ganglia. In order to assess if these differences were due to different kinetics of the CO_2_ response we compared the shape and specifically the on- and offset dynamics of the response of PCA variants with controls and did not find any significant differences.

Altogether, these results suggest that the observed differences are due to a decreased amplitude of the CO_2_ response in the fPCA group. However, it should be noted that local differences in vasoreactivity vary substantially between studies [16]. Therefore, replication of this data specifically in a population of younger adults will be crucial. Effects of the vascular variant might be more pronounced in the elderly, as the ability to vasodilate and compensate is reduced in the elderly due to atherosclerosis and decreased vascular flexibility [17, 18].

Our finding that the CVR is reduced in the occipital and temporal cortex in fPCA participants underline that the watershed areas are affected by the type of supply of the PCA.

From a clinical viewpoint, the variation in the CVR maps observed in fPCA cases may be relevant in the context of cerebral artery stenosis. It is well known that there is an increased risk of micro-ischemic lesions during the peri-interventional period for internal carotid artery stenosis, thromboendarterectomy or stenting [5]. It was postulated that micro-emboli occur in the brain, yet in the presence of a normal perfusion pressure, these micro-emboli are washed-out and do not cause clinically apparent micro-infarcts [19]. Our results imply that the CVR is reduced in the presence of a fPCA, which might indicate that patients with a fPCA might have an even higher risk for peri-procedural micro-ischemic lesions, and that the lesion distribution might be modified with respect to patients with a normal anatomy of the circle of Willis. In the same line, the presence of a fPCA might increase the risk of developing the posterior reversible encephalopathy syndrome (PRES), a condition of critically ill patients with various underlying diseases, characterized by headache, nausea, seizures, and visual loss. PRES is related to impaired vascular auto-regulation and affects typically posterior and/or posterior watershed areas [20, 21]. Our study shows a decrease in vascular regulation when the PCA is supplied by the anterior instead of the normal posterior circulation therefore indicating a greater risk for PRES. This possibility is supported by the sometimes asymmetric manifestation of clinical PRES [20] that might be related to unilateral vascularization variants. Future clinical studies are needed to elucidate the contribution of fPCA variant in these pathologies.

### CBF

Concerning the comparison of CBF between the one-side vascular variant (left variants were mirrored to pool all variants together) versus control participants, we found non-significant variations present within the watershed area of the MCA and PCA. In the presence of a fPCA, the territories of ACA, MCA and PCA derive the blood supply from the anterior circulation, i.e. only one vessel, notably the internal carotid artery. In contrast, in the classic configuration, the ACA and MCA territories are supplied by the internal carotid artery, while the PCA territory is supplied by the vertebro-basilar system, i.e. two supplying vessels. This difference in arterial supply via one versus two arterial systems might explain a mild reduction of the cerebral perfusion at baseline in the presence of a fPCA.

On the other hand, this could be explained by a methodological limitation of ASL in general. The single TI ASL that we used is optimized to measure CBF at the time of maximal labeled CBF for a normal vascularization. However, a slightly increased perfusion transit time for the fPCA subjects (as reported by [2]) would shift the peak of the maximum labeled CBF to a later time point. Consequently, the resulting relCBF map could under-estimate the real cerebral perfusion. Future studies in large series of fPCA subjects are needed to exclude the possibility that the PCA variant may have an influence on the CBF, in particular within the watershed areas.

### Functional Connectivity

Our results also indicate that the fPCA variant has little impact on BOLD resting-state functional connectivity. These results suggest that differences observed in FC are not biased by the type of PCA. It has previously been shown that perfusion variables and resting-state fMRI are not related in a linear way [22, 23] so that it is not surprising that we find small differences in the ASL data without FC differences in the BOLD data.

### Limitations

Since we corrected for false positive errors only, we can conclude that there were no significant differences in the sFC or CBF. Future studies could additionally estimate sensitivity at the expense of additional assumptions of an explicit alternative hypothesis as well. Moreover, we used the CO2 challenge for resting-state functional connectivity analysis as well after regressing out the CO2 challenge. However, we cannot exclude that some more subtle or long-lasting effects of CO_2_ administration might have confounded theses results. A replication study with pure resting-state data would therefore be desirable.

In addition, our fPCA group was rather small in comparison to the control group and further studies that specifically target fPCA subject recruitment could increase the statistical power of tests conducted to detect differences.

Last but not least, this series includes elderly, healthy adults who are clearly not representative of the general population.

## Conclusion

In conclusion, the presence of a fetal-type PCA impacts the response to CO_2_ and might also impact the cerebral blood flow. This indicates that studies looking at perfusion variables should take into account the vascularization. In contrast, conventional functional connectivity does not seem to be altered by the vascularization of the PCA.

## Supporting Information

S1 File Differences in the CO_2_ response between Controls and unilateral fetal PCA (not thresholded, fixed effects analysis, NIfTI file). (NII)

S2 File Differences in the CO_2_ response between Controls and unilateral fetal PCA (p<0.05, fixed effects analysis, NIfTI file). (NII)

S3 File Differences in baseline cerebral blood flow between Controls and unilateral fetal PCA (not thresholded, TFCE-corrected, NIfTI file). (NII)

S4 File F statistic values of the difference between vascular variants and controls (ANOVA test) for each connection (AAL parcellation, matlab file). (MAT)

S5 File F statistic values of the difference between vascular variants and controls (ANOVA test) for each connection (vascular territory parcellation, matlab file). (MAT)

S6 File Differences in baseline cerebral blood flow between unilateral fetal PCA and Controls (not thresholded, TFCE-corrected, NIfTI file). (NII)
